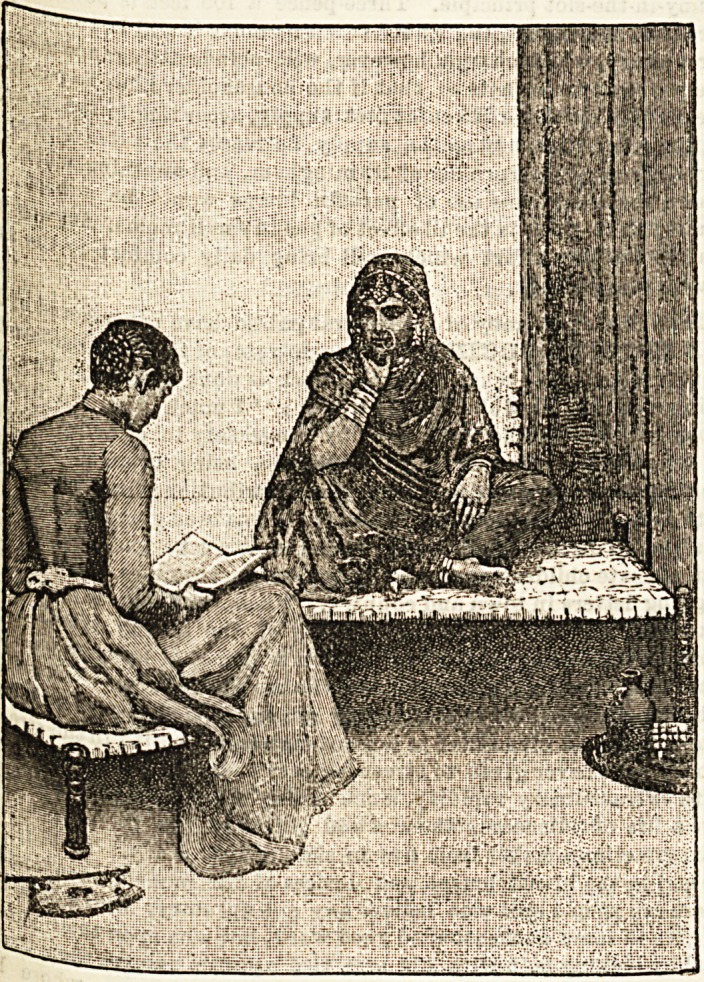# Zenana Bible and Medical Mission

**Published:** 1890-09-27

**Authors:** 


### Zenana Bible and Medical Mission.

There are now forty millions of native women shut up in the Indian Zenanas, and these may only be visited by others of their sex. The society sends earnest female missionaries among them, who are also qualified in certain branches of medicine, and thus two useful functions are combined. It is also unsectarian, and co-operates with all Evangelical Protestant Missionary Societies in India. It is hoped to build a hospital at Patna, the cost of which will be about £2,000. Of this, £1,000 is already forthcoming, and the society now appeal for the balance. Six additional Lady Missionaries are also wanted for six large centres of population, and a special effort is in progress to raise the income to £20,000 per annum. The attendances at hospitals last year exceeded 15,000, exclusive of in-patients, each institution being presided over by fully qualified lady doctors. The work has doubled during the last decade, the following being the agencies it employs: (1) Normal Schools, where Christian training is given to European,

Eurasian, and native young women, to qualify them as Teachers, etc. (2) Zenana Missionary Ladies, sent out from England to superintend the various branches of work carried on at the stations; Zenana Visitation: Girls' Schools; Village work, &c. (3) Medical Missionary Ladies and Native Nurses; the former, fully qualified Medical Practitioners, whose special Mission is to alleviate, in sickness, the sufferings of the Women of India, and at the same time administer to their spiritual need. (4) Female Vernacular Schools for Hindu and Mohammedan Children, in which religious instruction has the first place. (5) Bible Women. Native Christian Women of suitable age and consistent character, who read and explain the Scriptures to their countrywomen in hospitals, jails, villages, and houses. It employs 61 European Missionaries and Eurasian Assistants, 125 Native Christian Teachers, Nurses; 58 Bible Women; and it possesses 63 Schools with 2,379 pupils, and 3 Normal Schools with 130 students training for Mission work. Its Missionaries have access to 1,379 Zenanas and private houses, with 1,829 pupils under Christian instruction; whilst its Bible Women visit periodically 1,190 private houses, and over 400 villages where they read and explain the Scriptures. Hon. Finance Secretary, W. T. Paton, Esq., 2, Adelphi-terrace, W.C.

## Figures and Tables

**Figure f1:**